# *Postpartum* acute renal failure: a multicenter study of risk factors in patients admitted to ICU

**DOI:** 10.1186/s13613-014-0036-6

**Published:** 2014-11-25

**Authors:** Marie Jonard, Anne-Sophie Ducloy-Bouthors, Eileen Boyle, Maryse Aucourt, Gaelle Gasan, Merce Jourdain, Virginie Mignaux, Nadia Tillouche, François Fourrier

**Affiliations:** 1 Servie de Réanimation Polyvalente, Centre de Réanimation, Hôpital Roger Salengro, CHRU, 2 Avenue Oscar Lambret, Lille Cedex 59037, France; 2 Service d’Anesthésie Obstétricale, Hôpital Jeanne de Flandre, CHRU, 2 Avenue Oscar Lambret, Lille Cedex 59037, France; 3 Royal Cancer Hospital, The Institute of Cancer Research, 123 Old Brompton Road, London SW7 3RP, UK; 4 Service de Réanimation Polyvalente, Centre Hospitalier du Docteur Schaffner, 99, route de la Bassée, Lens 62300, France; 5 Service de Réanimation polyvalente, Centre Hospitalier de Valenciennes, Avenue Désandrouin, Valenciennes 59300, France; 6 Service de Maternité Monaco, Centre Hospitalier de Valenciennes, Avenue Désandrouin, Valenciennes 59300, France

**Keywords:** Pregnancy, Postpartum complications, Intensive care, Acute renal failure, HELLP syndrome, Postpartum haemorrhage, Hyperoncotic albumin, Tranexamic acid

## Abstract

**Background:**

Even in developed countries, severe specific pregnancy complications may occur in the immediate *postpartum* period and require admission to the ICU. The characteristics and risk factors of acute renal failure (ARF) induced by these complications and their treatments are not well known.

**Methods:**

We performed a retrospective multicenter study in three intensive care departments linked to level III maternity wards in the north of France. All patients admitted to ICU for *postpartum* complications over a 5-year period (2008 to 2012) were included. Clinical and biological data, delivery characteristics, type of complications, and treatments were compared by univariate and multivariate analyses according to the occurrence and severity of ARF.

**Results:**

One hundred eighty-two patients admitted to ICU for *postpartum* complications were included in the study. Sixty-eight patients (37%) developed an ARF: 49 with a low or medium severity and 19 with a severe ARF requiring renal replacement therapy. Hemolysis, elevated liver enzyme, and low platelet count (HELLP) syndrome on its own (*p* = 0.047) or combined with *postpartum* haemorrhage (*p* = 0.003), previous treatment by hyperoncotic albumin infusion (*p* = 0.001) and blockade of fibrinolysis by tranexamic acid (*p* = 0.03), was associated with secondary ARF. By multivariate analysis, the only independent factors were the association of HELLP syndrome with *postpartum* haemorrhage and the use of hyperoncotic albumin infusion.

**Conclusions:**

HELLP syndrome associated with *postpartum* haemorrhage induces a high risk of ARF in the complicated *postpartum* setting. A particular attention should be given to treatments that could worsen the kidney function in that situation.

## Background

Maternal mortality has significantly decreased over the last decades. Nevertheless, even today, the short period that immediately follows childbirth may be complicated thus putting the mother's life at stake. In the whole pregnant population, approximately 0.2% of women will require critical care, and this percentage can reach 1% in medium and low resource countries [1],[2]. The main causes of intensive care unit (ICU) admission are *postpartum* hemorrhage, preeclampsia and related diseases, and sepsis [3],[4].

Acute renal failure (ARF) is rare in that setting. Its incidence has decreased from 1/3,000 to 1/20,000 between the 1960's and today [5]. Nevertheless, it remains an important source of morbidity and mortality. ARF is more often due to pregnancy-related diseases. The first peak of incidence, during the first trimester, is dominated by infection and illegal abortion in low-resource countries. The second peak occurs during the third trimester and is related to preeclampsia, the hemolysis, elevated liver enzyme, and low platelet count (HELLP) syndrome, acute fatty liver disease of pregnancy, or *postpartum* hemorrhage.

Classically, it is believed that ARF occurs in 1% of severe preeclampsia, 3% to 15% of HELLP syndromes [6],[7] and 60% in acute fatty liver disease of pregnancy [8], with preeclampsia and HELLP syndrome covering 40% of all cases [9]. However, it is not clear from clinical studies whether the coagulopathy induced by the HELLP syndrome could increase the magnitude of *postpartum* hemorrhage or, conversely, whether uterine hemorrhage could aggravate the thrombotic microangiopathy of the HELLP syndrome and subsequent renal ischemia. Their association might represent a peculiar risk of ARF in the *postpartum* setting.

The aim of our study was to identify the risk of ARF induced by *postpartum* hemorrhage, preeclampsia, HELLP syndrome, and their association in patients admitted to the ICU in the *postpartum* period. We also tried to document in this setting the possible impact of the treatments used to control hemorrhage and arterial hypertension.

## Methods

We performed a multicenter observational study over a 5-year period in three intensive care departments attached to level III maternity wards in the north of France. In the geographic area of these three hospitals, obstetrical care is strictly organized, and women developing severe *postpartum* complications are transferred to these three referral centers comprising a level III maternity ward and one ICU.

**Figure 1:**
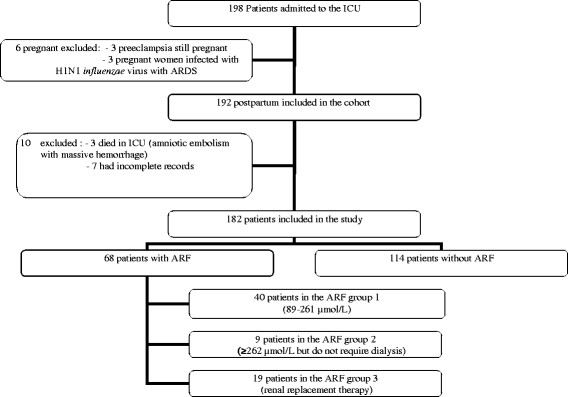
Flowchart of the cohort.

All patients admitted to the ICU immediately after childbirth were included in our study. Patients admitted to the ICU during pregnancy or admitted for non-pregnancy related disease were excluded (see flow chart, Figure 1). Case notes were retrospectively analyzed. All observations were reviewed anonymously. Therefore, informed consent was not sought for in accordance to the French clinical research guidelines.

### Definitions and criteria

The main objective of our study was to compare women with ARF and those without to document the risk factors of ARF in the complicated *postpartum* period.

ARF was diagnosed when serum creatinine levels were 89 μmol/L or greater, from delivery until the sixth day *postpartum*. Patients were subdivided into the following three groups based on the highest value of serum creatinine and on renal replacement therapy requirement:

 The ARF 1 group, with serum creatinine levels between 89 and 261 μmol/L;

 The ARF 2 group, when serum creatinine was greater or equal to 262 μmol/L but patients did not require renal replacement therapy; and

 The ARF 3 group, when patients required renal replacement therapy.

The risk, injury, failure, loss of function, and end-stage renal disease (RIFLE) score was also calculated on admission and discharge for follow-up [10],[11]. Medium and long-term outcome (1 to 5 years) was determined via the North of France Kidney Registry related to pregnancy [Néphronor* registry] and via the patient's general practitioner.

*Postpartum* hemorrhage was diagnosed when blood loss in the first 24 h after delivery was greater than 500 mL following vaginal delivery or 1,000 mL following cesarean section [12].

 Preeclampsia was diagnosed if blood pressure was ≥140/90 mmHg and proteinuria ≥300 mg/day at anytime from week 20 of gestation [13]. HELLP syndrome was defined by the combination of thrombocytopenia (<100 G/L), elevated liver enzymes (AST >70 UI/L), and hemolysis [14]. Hemolysis was defined by a bilirubin >12 mg/L, LDH >600 UI/L, haptoglobin <0.5 mg/L, and the presence of more than 0.5% schizocytes. In patients who presented a *postpartum* hemorrhage and/or needed platelet transfusion, the decrease in platelet count was not used as diagnostic criteria of HELLP syndrome, except when the HELLP syndrome had clearly preceded the hemorrhagic complication.

 Criteria for diagnosis of acute fatty liver disease of pregnancy include six or more of the following (Swansea criteria): vomiting, abdominal pain, polydipsia/polyuria, encephalopathy, elevated bilirubin (>14 μmol/L), hypoglycemia (<4 μmol/L), elevated blood uric acid (>340 μmol/L), leukocytosis (>11 × 10^9^/L), ascites or bright liver on ultrasound scan, elevated transaminases (aspartate aminotransferase or alanine aminotransferase >42 IU/L), elevated ammonia (>47 μmol/L), renal impairment (creatinine >150 μmol/L), and coagulopathy (prothrombin time >14 s or activated partial thromboplastin time >34 s)[15].

 The possible occurrence of kidney hypoperfusion was considered when mean arterial pressure was lower than 60 mmHg on two separate occasions between delivery and the fourth day after. Patients receiving vasoactive support (norepinephrine) after severe *postpartum* haemorrhage were considered hypotensive.

 The diagnosis of severe sepsis used the definitions of the American College of Chest Physicians and the Society of Critical Care Medicine [16].

 The patient's severity was assessed by the SAPS II score during the first 24 h after admission in the ICU [17].

 The severity of coagulopathy was assessed by the International Society of Thrombosis and Haemostasis (ISTH) disseminated intravascular coagulation (DIC) score [18]. Due to an expected low number of patients developing overt DIC, an ISTH score of >3 was considered as witness of severe coagulation disorders.

 All drugs received from delivery until the fourth day *postpartum* were considered with special attention given to the use and amount of the following:

 Plasma volume expanders: fluids (crystalloids, artificial colloids, 4% and 20% hyperoncotic serum albumin), blood products (packed red cells, platelets, fresh plasma, and fibrinogen)

 Antifibrinolytic: tranexamic acid

 Anti-hypertensive drugs, diuretics, angiotensin-converting enzyme (ACE) inhibitors

 Nephrotoxic agents: aminoglycosides and radio-contrast media.

### Statistical analysis

Statistical analysis was performed using SAS software (SAS Institute, Cary, NC, 25513; version 9.3). Results are given as number, mean, median, and interquartile range for quantitative variables, and percentages for nominal variables.

Comparisons between groups were done using Student's *t-*test and Mann-Whitney's *U* test when appropriate. When more that two groups were present, a Kruskal-Wallis one-way analysis of variance was performed. Comparisons were performed using either a chi-squared test or Fisher's exact test when appropriate.

First of all, clinical and biological characteristics were compared according to the occurrence of ARF. Regarding pregnancy-related diseases, the frequency of ARF was compared according to the development of preeclampsia, HELLP syndrome, and *postpartum* hemorrhage and to their association (preeclampsia + *postpartum* hemorrhage; HELLP + *postpartum* hemorrhage). Differences in all recorded variables were compared by univariate analysis. Then, to isolate independent risk factors of ARF, all variables identified in the univariate analysis with a *p* <0.2 and less than 20% missing data were included in a multivariate analysis followed by a bootstrap analysis. Odd ratios for ARF and their 95% confidence intervals (95%) were determined with *p* <0.05 taken as the level of significance.

## Results

**Figure 2:**
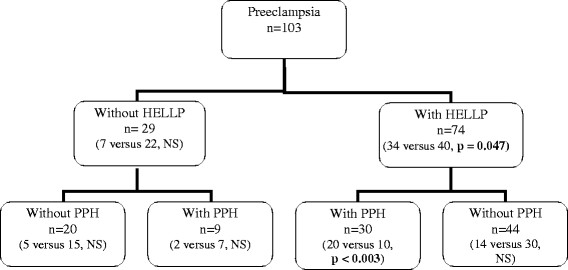
**Flowchart of the 103 patients admitted in the ICU for preeclampsia.** Data are given in number. Numbers in bracket represent the number of patients with ARF versus patients without ARF and the *p* value between ARF and no ARF group. PPH: *postpartum* hemorrhage.

During the 5 years of the study, 198 patients were admitted to ICU in the *postpartum* period, with 182 included in the study (see flowchart in Figures 1 and 2). During the same period, 59,302 deliveries were included in the pregnancy registry of the three geographic areas. Therefore, patients admitted to the ICU for *postpartum* complications represented 0.33% of all deliveries in our study. Main demographic data and causes of ICU admission are given in Table 1 and Figure 2, respectively.

**Table 1:** Patients' baseline characteristics

	**All patients** ***n*** **= 182**	**ARF group** ***n*** **= 68**	**No ARF group** ***n*** **= 114**	***p*** **value**
Age, years^a^	29 [26 to 34]	29 [26 to 33]	30 [25 to 34]	0.857
Number of pregnancy^a^	2 [1 to 3]	2 [1 to 3]	2 [1 to 3]	0.4
Parity^a^	1 [1 to 3]	1 [1 to 2]	1 [1 to 3]	0.8
Gestational diabetes	25 (14)	10 (15)	15 (13)	0.7
Chronic hypertension	6 (3)	3 (4)	3 (3)	NA
Gestational hypertension	13 (7)	5 (7)	8 (7)	0.27
Obesity	26 (14)	14 (21)	12 (11)	0.17
Twin gestation	11 (6)	6 (9)	5 (4)	0.33
Term^a^ (week of gestation)	37 [33 to 39]	35 [33 to 39]	37 [32 to 39]	0.82
Antihypertensive agents before pregnancy	4 (2)	1 (1)	3 (3)	NA
Antihypertensive agents during pregnancy	41 (23)	14 (21)	27 (23)	NA
Cesarean delivery	128 (71)	45 (62)	83 (76)	0.15
Vaginal childbirth	53 (29)	23 (34)	30 (26)	0.14
SAPS II score^a^	21 [14 to 31]	28 [17 to 36]	18 [13 to 24]	***<*** 0.001
Length of stay in the ICU (days)^a^	3 [2 to 4]	4 [3 to 7]	2 [2 to 3]	***<*** 0.001

Data are *N* (%) for qualitative variables. ^a^Quantitative variables are presented as median [interquartile range: 25th to 75th percentiles]. NA = not applicable; SAPS: simplified acute physiology score.

### Classification and outcome of ARF (main results are presented in Table 2 )

**Table 2:** Classification of ARF and outcome

	**ARF 1** ***n*** **= 40**	**ARF 2** ***n*** **= 9**	**ARF 3** ***n*** **= 19**	**All ARF** ***n*** **= 68**	***p*** **value**
*RIFLE* first day in ICU					**<**0.0001
*RISK*	20 (50)	0	0	20 (29)	
*INJURY*	13 (32)	0	0	13 (19)	
*FAILURE*	7 (17)	9 (100)	19 (100)	35 (51)	
RIFLE last day in ICU					NA
*RISK*	5 (12)	0	0	5 (7)	
*INJURY*	2 (5)	0	0	2 (3)	
*FAILURE*	1 (2)	7 (78)	18 (95)	26 (38)	
Normalized renal function in the ICU	32 (80)	2 (22)	1 (5)	35 (51)	
Transfer to nephrology ward	1 (2)	4 (44)	16 (84)	21 (31)	**<**0.0001
Delay of renal recovery					NA
<15 days	34 (85)	2 (22)	1 (5)	37 (54)	
15 days to 1 month	5 (12)	3 (33)	1 (5)	9(13)	
1 month to 6 months	1 (2)	3 (33)	7 (36)	11 (16)	
>6 months	0	0	3 (16)	4 (6)	
>1 year or chronic renal failure	0	1 (11)	7 (37)	8 (12)	
Chronic hemodialysis	0	1 (11)	2 (10)	3 (4)	NA

Data are *N* (%). NA = not applicable.

ARF complicated the course of 68 patients (37.3%). Among these 68 patients, 40 (59%) had a low-grade ARF (ARF 1), 9 (13%) a medium grade (ARF 2), and 19 (28%) a high grade (ARF 3) requiring renal replacement therapy.

According to the RIFLE classification, 20 patients in the ARF 1 group had a RISK score on admission and had a normal renal function on discharge. The remainder of the group was considered as INJURY or FAILURE. They all evolved favorably before discharge. Overall, 85% (*n* = 34) had a normal kidney function within 2 weeks. Fifteen patients (12%) normalized their renal function within 1 month and one patient had a delayed recovery (6 months).

In the ARF 2 group, the nine patients were all classified as FAILURE according to RIFLE. On discharge, two had completely recovered and seven still had a FAILURE score. Among those, three normalized their function within one month and three in 6 months. One patient ended up with chronic kidney disease requiring dialysis.

In the ARF 3 group, except one patient who normalized her renal function while in the ICU, the 19 patients were considered as FAILURE on admission and on discharge. The majority of them normalized their kidney function between 6 to 12 months. Seven (37%) went on to chronic dialysis. In these seven cases, cortical necrosis was diagnosed by kidney biopsy and/or magnetic resonance imaging after discharge from the ICU to the nephrology ward.

No case of atypical HUS was observed in our patients.

### *Postpartum* complications associated with ARF

Patient's characteristics and treatments are summarized in Tables 3, 4, and 5 and Figure 2.

**Table 3:** Frequency or acute renal failure according to the type of pregnancy and delivery complications

	**All patients** ***n*** **= 182**	**ARF group** ***n*** **= 68**	**No ARF group** ***n*** **= 114**	***p*** **value**
PE without HELLP	20 (11)	5 (7.3)	15 (13.1)	0.225
Eclampsia	21 (12)	5 (7)	16 (14)	0.172
AFLP	5 (3)	4 (6)	1 (1)	NA
Isolated HELLP without PPH	44 (24)	14 (20.5)	30 (26.3)	0.382
Isolated PPH	70 (39)	21 (31)	49 (43)	0.104
Volume of bleeding^a^ (mL)	2,500 [1,525 to 4,000]	2,000 [1,500 to 3,000]	3,000 [1,750 to 4,050]	0.035
PPH + PE without HELLP	9 (5)	2 (2.9)	7 (6.1)	0.335
PPH + HELLP	30 (17)	20 (29)	*10 (9)*	0.0003

Data are *N* (%) for qualitative variables. ^a^Quantitative variables are presented as median [interquartile range: 25th to 75th percentiles]. NA = not applicable; AFLP: acute fatty liver pregnancy; PE: preeclampsia; HELLP: hemolysis, elevated liver, and low platelet count syndrome; PPH: *postpartum* hemorrhage.

**Table 4:** **Comparisons according to the severity of ARF (*n*** **= 68)**

	**ARF 1** ***n*** **= 40**	**ARF 2** ***n*** **= 9**	**ARF 3** ***n*** **= 19**	***p*** **value**
Age, years^a^	30 [26 to 34]	25 [21 to 28]	31 [27 to 33]	0.18
Term (week of gestation)^a^	37 [33 to 39]	33 [29 to 38]	37 [36 to 39]	0,2
Number of pregnancy^a^	1 [1 to 2]	1 [1 to 1]	2 [1 to 3]	*0.04*
Twin gestation	2 (5)	0	4 (21)	0.087
SAPS II score^a^	20 [16 to 29]	27 [21 to 29]	37 [33 to 45]	***<*** 0.001
Length of ICU stay (days)^a^	3.5 [2 to 4.5]	5 [3 to 5]	9 [5 to 13]	***<*** 0.001
All PE; *n* = 41	24 (60)	5 (56)	12 (63)	0.93
Whose HELLP; *n* = 34	19 (47)	4 (44)	11 (58)	0.77
PPH; *n* = 43	25 (62)	3 (33)	15 (79)	0.064
Volume of bleeding (mL)^a^	2,300 [1,600 to 3,000]	1,500 [750 to 4,000]	2,000 [1,500 to 3,000]	NA
PPH only; *n* = 21	12 (30)	2 (22)	7 (37)	0.72
Hypotension; *n* = 11	7 (17)	0	4 (21)	0.46
PPH + HELLP; *n* = 20	11 (27)	1 (11)	8 (42)	0.22
ISTH score >3; *n* = 29	17 (46)	2 (29)	10 (67)	0.23

Data are *N* (%) for qualitative variables. Incidence is given in brackets considering missing data. About treatment, the incidence is related to the number of patients who actually received the treatment. ^a^Quantitative variables are presented as median [space interquartile range: 25th to 75th percentiles]. NA = not applicable; SAPS: simplified acute physiology score; PE: preeclampsia; HELLP: hemolysis, elevated liver, and low platelet count syndrome; AFLP: acute fatty liver pregnancy; ISTH = International Society on Thrombosis and Hemostasis score.

**Table 5:** Frequency of ARF according to the type and dose of treatments

	**ARF group** ***n*** **= 68**	**No ARF group** ***n*** **= 114**	***p*** **value**
Crystalloids			
*n* = 121	52 (93)	69 (84)	0.12
mL^a^	2,000 [1,500 to 3,000]	2,000 [1,000 to 2,500]	0.58
Artificial colloids			
*n* = 100	40 (74)	60 (71)	0.7
mL^a^	1,000 [1,000 to 1,750]	1,000 [1,000 to 2,000]	0.66
Hyperoncotic albumin			
*n* = 32	20 (29)	12 (10)	0.0012
mL^a^	250 [200 to 400]	200 [200 to 300]	0.52
Albumin 4%			
*n* = 24	8 (12)	16 (14)	0.66
mL^a^	750 [500 to 1,375]	750 [500 to 1,000]	0.94
Packed red cells			
*n* = 107	44 (65)	63 (56)	0.26
mL^a^	800 [600 to 120]	1,000 [600 to 1,600]	0.11
Platelets concentrates			
*n* = 68	25 (38)	43 (38)	0.94
*n*^a^	1 [1 to 2]	1 [1 to 2]	0.55
Fresh frozen plasma (FFP)			
*n* = 94	37 (56)	57 (51)	0.54
*n*^a^	4 [3 to 6]	5 [3 to 7]	0.11
Fibrinogen concentrate			
*n* = 107	45 (66)	62 (57)	0.24
g^a^	4.50 [3 to 7.5]	5.5 [3 to 7.5]	0.75
Activated factor VII			
*n* = 7	4 (6)	3 (3)	NA
Tranexamic acid			
*n* = 79	37 (54)	42 (38)	0.03
g^a^	5 [4 to 7]	5 [3 to 9]	0.66
Antihypertensive drugs in ICU			
*n* = 101	43 (64)	58 (52)	0.10
*n*^a^	1 [0 to 3]	1 [0 to 2]	0.03
Diuretics			
*n* = 73	36 (53)	37 (32)	0.006
ACE inhibitors			
*n* = 50	24 (35)	26 (23)	0.079
Radio-contrast media			
*n* = 27	11 (16)	16 (14)	0.71
Aminoglycosides			
*n* = 33	12 (18)	21 (19)	0.87

Data are *N* (%) for qualitative variables. Frequency is given in brackets considering missing data. ^a^Quantitative variables are presented as median [space interquartile range: 25th to 75th percentiles]. mL: milliter; *n*: number; g: grams; NA = not applicable.

The frequency of ARF was different according to the type of *postpartum* complications. When these complications were not associated, the frequency of ARF reached 24% in patients with preeclampsia, 30% in *postpartum* hemorrhage, 46% in HELLP, and 4 of 5 with acute fatty liver disease of pregnancy. In the high severity ARF groups 2 and 3, nine patients among 28 had presented an isolated *postpartum* hemorrhage and nine the association HELLP-*postpartum* hemorrhage. The univariate analysis showed that when isolated, preeclampsia, HELLP syndrome, and *postpartum* hemorrhage were not significantly linked to ARF.

Thirty patients developed the association HELLP syndrome-*postpartum* hemorrhage. Complete data of this subgroup are given in Table 6. The frequency of ARF was significantly higher in patients who had presented this association (frequency of ARF: 67%; *p* = 0.003). In two cases only, the HELLP syndrome occurred in the 48 h after the hemorrhagic event. In 28 cases, *postpartum* hemorrhage occurred sequentially after completion of the HELLP syndrome. The amount of *postpartum* bleeding was not higher in these patients with even a trend to a lower amount of bleeding (median 2,000 mL in isolated *postpartum* hemorrhage vs 1,700 mL when *postpartum* hemorrhage was associated to HELLP; *p* = 0.18).

**Table 6:** Characteristics and comparisons of the 30 patients with HELLP associated to PPH

	**ARF group** ***n*** **= 20**	**No ARF group** ***n*** **= 10**	***p*** **value**
Baseline characteristics			
Age, years^a^	32 [27 to 35]	29 [24 to 32]	0.1
Number of pregnancy^a^	2.5 [1 to 3]	1.5 [1 to 3]	0.45
Parity^a^	2 [1 to 3]	1 [1 to 2]	0.45
Term^a^ (weeks of gestation)	37 [36 to 39]	37 [32 to 38]	NA
Twin gestation *n* = 3	2 (10)	1 (10)	NA
Obesity *n* = 8	6 (30)	2 (20)	NA
Chronic hypertension *n* = 2	2 (10)	0	NA
Gestational hypertension *n* = 4	4 (20)	0	NA
Gestational diabetes *n* = 8	6 (30)	2 (20)	NA
Antihypertensive drugs before pregnancy *n* = 1	1 (5)	0	NA
Antihypertensive drugs during pregnancy *n* = 9	6 (30)	3 (30)	NA
SAPS II score^a^	31 [22 to 39]	15 [12 to 23.5]	0.01
Length of stay in the ICU^a^ (day)	5.5 [4 to 10]	3 [2 to 4]	0.04
Complications of delivery			
PPH secondary to HELLP *n* = 28	18 (90)	10 (100)	0.3
*Postpartum* HELLP secondary to PPH *n* = 2	2 (10)	0	NA
Volume of bleeding^a^ mL	1,600 [1,050 to 2,100]	2100 [975–3175]	0.6
Hypotension *n* = 7	4 (20)	3 (30)	0.4
ISTH >3 *n* = 17	11 (55)	6 (60)	0.4
Treatments			
Crystalloids			
*n* = 22	16 (80)	6 (60)	0.38
mL^a^	2,500 [1,500 to 3,000]	2,500 [2,000 to 2,625]	0.9
Artificial colloids			
*n* = 21	15 (75)	6 (60)	0.43
mL^a^	1,000 [1,000 to 1,500]	1,250 [500 to 1,625]	0.8
Hyperoncotic albumin			
*n* = 8	6 (30)	2 (20)	0.68
mL^a^	250 [200 to 400]	300 [300 to 400]	0.22
4% albumin			
*n* = 8	6 (30)	2 (20)	0.68
mL^a^	750 [437 to 1,812]	750 [500 to 750]	0.86
Packed red cells			
*n* = 24	16 (80)	8 (80)	NA
mL^a^	700 [450 to 1,150]	800 [450 to 1,150]	0.9
Platelets concentrates			
*n* = 14	9 (45)	5 (50)	0.8
*n*^a^	1 [1 to 1.5]	1 [1 to 2]	0.09
Fresh frozen plasma			
*n* = 16	10 (50)	6 (60)	0.6
*n*^a^	5 [2.7 to 6.2]	2.5 [2 to 4.2]	0.09
Fibrinogen concentrates			
*n* = 25	17 (85)	8 (80)	NA
g^a^	4.5 [3 to 8]	3 [2 to 7]	0.23
Activated factor VII			
*n* = 1	1 (5)	0	NA
Tranexamic acid			
*n* = 19	16 (80)	3 (30)	0.01
g^a^	5.5 [3.6 to 6.7]	5 [4 to 5]	0.6
Antihypertensive drugs in ICU			
*n* = 25	17 (85)	8 (80)	NA
Diuretics			
*n* = 16	12 (60)	4 (40)	0.3
ACE inhibitors			
*n* = 14	10 (50)	4 (40)	0.6
Radio-contrast media			
*n* = 3	0	3 (30)	NA
Aminoglycosides			
*n* = 1	1 (5)	0	NA

Data are *N* (%) for qualitative variables. Incidence is given in brackets considering missing data. Concerning treatments, the incidence is related to the number of patients who actually received the treatment. ^a^Quantitative variables are presented as median [space interquartile range: 25th to 75th percentiles]. mL: milliter; *n*: number; g: grams; NA = not applicable.

Concerning the severity of coagulation disorders, patients who developed ARF had more frequently an ISTH score greater than 3 (49 vs 32%; *p* = 0.034). However, in the ARF group, the median value of the ISTH score was not significantly different between patients with or without *postpartum* hemorrhage (median 3.0 vs 3.0 points; NS), with or without HELLP syndrome (3.0 ± 1.8 vs 2.7 ± 1.9; NS), and with or without their association (2.8 ± 1.5 vs 2.6 ± 2.0; NS). Among the 28 patients who developed an ARF grade 2 or 3, seven patients only had an ISTH score of >4, consistent with acute overt DIC. Due to this low number, an ISTH score of >3 was entered in the multivariate analysis to document the possible role of coagulation disorders.

Concerning treatments given between delivery and the fourth-day *postpartum*, the univariate analysis showed that patients in the ARF group received more frequently tranexamic acid (54% vs 38% in the non-ARF group; *p* = 0.03), diuretics (*p* = 0.006), and concentrated serum albumin (*p* = 0.0012) (Table 5). Of note, 83% of patients with *postpartum* hemorrhage in the ARF group received tranexamic acid (mean dose: 5 ± 4.84 g).

The amount of packed red cells, platelets, fibrinogen, and other plasma substitutes was not different. Seven patients received recombinant activated factor seven. Four of these seven patients presented an acute renal failure, one in the ARF group 1, one in the ARF group 2, and two in the ARF group 3.

In the multivariate analysis of risk factors for ARF, the following variables were not significant: *postpartum* hemorrhage, preeclampsia, HELLP, ISTH of >3, and use of tranexamic acid. The only independent risk factors identified were the association of *postpartum* hemorrhage with the HELLP syndrome (OR [CI 95%] = 4.1 [1.75 to 9.66]; *p* = 0.0012) and the administration of 20% hyperoncotic serum albumin (OR [CI 95%] = 3.34 [1.47 to 7.60]; *p* = 0.0039).

## Discussion

In our study, ARF occurred in 37% of patients admitted to ICU and 29% of them required dialysis. In 12% of the cases, ARF evolved into chronic kidney disease due to cortical necrosis. The frequency of ARF clearly depends on the cutoff level of blood creatinine used to define renal failure. We chose in our study a cutoff blood creatinine level of 89 μmol/L according to French recommendations [19]. During pregnancy, physiological changes in renal blood flow and glomerular filtration rate induce a lower level of blood creatinine so that cutoff levels of the general population are not reliable. A worldwide definition of criteria in pregnant women could help to better compare studies of ARF. Our study certainly overestimates the incidence of severe cases of ARF as we focalized on an ICU setting. However, an increase in the incidence of severe ARF in developed countries was recently documented in studies from Canada [20]. It might be related to demographic changes such as increased age, obesity, caesarian section [21], incidence of preeclampsia [22] or *postpartum* hemorrhage, and their treatments. This was also documented in two recent studies in Australia and Canada [23],[24].

Our data suggest that *postpartum* hemorrhage and HELLP syndrome are common causes of ARF and their combination an independent risk factor of its occurrence. Gurrieri et al. identified 55 cases of ARF over a 5-year period, seven of which were related to HELLP syndrome [25]. Sibai et al. in 1993 documented an incidence of ARF of 7.4% among HELLP syndrome patients, 31% of which required dialysis [26], but the association of *postpartum* hemorrhage with the HELLP syndrome was not documented in these studies.

Our study provides evidence that HELLP syndrome in combination with *postpartum* hemorrhage is an important factor for occurrence of ARF. The pathophysiologic mechanisms of ARF in that situation could not be documented in our study, except in women who did not recover and developed a cortical necrosis. Usually, acute tubular necrosis is considered the most frequent histological lesion found in women suffering from HELLP syndrome and acute kidney injury. In our study, the HELLP syndrome mostly preceded the *postpartum* hemorrhagic complication. It can be hypothesized that the HELLP-induced thrombotic microangiopathy and hemolysis could induce a first shot phenomenon of tubular ischemia, sequentially aggravated by *postpartum* haemorrhage. However, we were unable to document that the sequential combination induced a more frequent arterial hypotension or a higher amount of blood loss that could aggravate kidney hypoperfusion. The first point should be considered with caution since in our study we used a cutoff level of 60 mmHg to define arterial hypotension. Recent guidelines have defined a higher a 70 mmHg level to document arterial hypotension in pregnant women. Regarding the role of acute hemorrhage in the occurrence of ARF, there was even a trend to lower blood loss in women suffering from HELLP syndrome and *postpartum* hemorrhage compared to isolated *postpartum* hemorrhage.

We also tried to document the potential role of coagulation disorders in the occurrence of ARF. As measured by the ISTH score, the incidence of overt DIC was low and there was only a trend to higher ISTH score in patients combining *postpartum* hemorrhage and HELLP syndrome. From a theoretical point of view, except in overt DIC, the procoagulant state induced by the HELLP syndrome could reduce the amount of hemorrhage. Due to the low number of cases, this hypothesis cannot be documented in our study. It seems more likely that the addition of thrombotic microangiopathy induced by the HELLP syndrome with hypovolemia and vasoconstriction induced by hemorrhage could ‘synergistically’ compromise both placental and kidney perfusion.

Our study was the first to analyze the impact of treatment. Hyperoncotic serum albumin was associated with ARF whereas 4% albumin had no impact. This is in accordance with previously published data in other settings. Preeclampsia may induce a hypovolemic state caused by hypoalbuminemia and capillary permeability dysfunction. The degree of hypovolemia increases significantly at the ascitis state. Albumin is often used in that situation although it has never demonstrated any benefit in terms of maternal or fetal morbidity [27],[28]. Outside of pregnancy, the use of hyperoncotic albumin seemed associated with development of acute renal failure [29]. The inferred mechanisms are the decrease in filtration pressure and a direct nephrotoxicity. Further studies are required to clarify that point in the *postpartum* setting.

Regarding haemostatic treatments used for *postpartum* hemorrhage, ARF was significantly associated to tranexamic acid in the univariate analysis. This could be due to a bias, since most patients whose *postpartum* course was complicated by *postpartum* hemorrhage received the inhibitor. From a physiological point of view, pregnancy is characterized by the progressive development of a pro-coagulant and antifibrinolytic state. Delivery is characterized by an acute and transient fibrinolysis due to the loss of the placental inhibitor of plasminogen activator and exacerbated by placental ischemia or uterine atony. Tranexamic acid binds in an irreversible fashion a lysine residue on plasminogen, preventing it from binding fibrin and tissue-plasminogen activator. Thus, blockade of fibrinolysis by tranexamic acid is used to reduce the severity and duration of *postpartum* hemorrhage. The possibility must also be considered that by increasing intravascular coagulation and renal ischemia, inhibition of fibrinolysis may lead to ARF and cortical necrosis in these situations. From a clinical perspective, the prophylactic use of tranexamic acid has been shown to reduce *postpartum* hemorrhage in three randomized clinical trials. Nevertheless, these studies were not powered to detect its impact on mortality, the frequency of hysterectomy, and thromboembolic complications [30]. There is no study regarding the use of tranexamic acid in a curative intent and its possible adverse effects on renal function. The possibility must be considered that adding insult to injury, antifibrinolytic drugs given to patients with a preexisting activated endothelium by HELLP syndrome may increase the risk of renal failure. In our study, 80% of the 20 patients who developed an ARF after the sequential occurrence of HELLP syndrome and *postpartum* hemorrhage had received tranexamic acid vs 30% only of those who did not develop ARF. Additionally, seven among the eight patients who developed chronic renal failure and cortical necrosis had received tranexamic acid. The incidence of cortical necrosis was evaluated by Leidmer et al. [31] to approximately 1/80,000 whereas our study has identified seven cases out 182, proved by renal biopsy or MRI over a 5-year period (3.8%). Recently, 12 cases of cortical necrosis following *postpartum* hemorrhage treated with tranexamic acid and associated with HELLP syndrome were reported by nephrologists to alert the national health authorities. [32]. In our study, the use of tranexamic acid was not documented as an independent risk factor in the multivariate bootstrap analysis, but taken together, these findings suggest that inhibition of fibrinolysis should be used with caution in women developing *postpartum* hemorrhage. The WOMAN study currently ongoing will assess the impact of tranexamic acid on the reduction of *postpartum* hemorrhage related to mortality and hysterectomy and give us additional information regarding the renal adverse effects of this drug [33].

### Limits of our study

Our multicenter study was conducted in only three ICUs in the north of France and included a limited number of cases thus hindering interpretation of data. All our patients were admitted in tertiary-care ICU and severely ill. This may introduce bias in the reported incidence and outcome of complications. It is highly possible that we could miss some patients developing an acute and rapidly resolving mild renal failure in the *postpartum* period.

Additionally, given the nature of this study, we were only able to identify factors strongly associated with ARF and our study could not document the cause-effect relationships between these factors and ARF. However, in this quite homogeneous population, we could perform an independent and exhaustive analysis of *postpartum* complications and of the potential role of treatments.

## Conclusions

Our study suggests that among pregnancy-related diseases, HELLP syndrome, when associated with *postpartum* hemorrhage, induces the highest risk of ARF. Our data do not support the possibility that this could be due to higher blood loss or more severe coagulation changes induced by the association. In these predisposed patients, some treatments such as hyper-oncotic serum albumin may be damageable for kidney function. A strong attention should be given to kidney function and the use of treatments at risk should be avoided.

## Competing interests

The authors declare that they have no competing interests.

## Authors’ contributions

All authors have made substantial contributions to this paper. MJ (corresponding author), FF, and MJ participated in the design of the study. MJ (corresponding author) conceived of the study and drafted the manuscript. MJ (corresponding author), AS, EB, GG, MA, VM, and NT participated in data recovery. FF participated in the design and coordination and helped to draft the manuscript. All authors read and approved the final manuscript.
